# Reporting Guidelines for Music-based Interventions checklist: explanation and elaboration guide

**DOI:** 10.3389/fpsyg.2025.1552659

**Published:** 2025-06-11

**Authors:** Sheri L. Robb, K. Maya Story, Elizabeth Harman, Debra S. Burns, Joke Bradt, Emmeline Edwards, Tasha L. Golden, Christian Gold, John R. Iversen, Assal Habibi, Julene K. Johnson, Miriam Lense, Susan M. Perkins, Stacey Springs

**Affiliations:** ^1^School of Nursing and School of Medicine, Indiana University, Indianapolis, IN, United States; ^2^School of Medicine and Richard L. Roundbush VA Medical Center, Indiana University, Indianapolis, IN, United States; ^3^College of Communication and Fine Arts, University of Memphis, Memphis, TN, United States; ^4^Department of Creative Arts Therapies, Drexel University, Philadelphia, PA, United States; ^5^National Center for Complementary and Integrative Health, Bethesda, MD, United States; ^6^Center for Arts in Medicine, University of Florida, Gainesville, FL, United States; ^7^NORCE Norwegian Research Centre AS, Bergan, Norway; ^8^Grieg Academy Department of Music, University of Bergan, Bergan, Norway; ^9^Department of Clinical and Health Psychology, Faculty of Psychology, University of Vienna, Vienna, Austria; ^10^Department of Psychology, Neuroscience and Behavior, McMaster University, Hamilton, ON, Canada; ^11^Brain and Creativity Institute, University of Southern California, Los Angeles, CA, United States; ^12^Institute for Health & Aging, University of California San Francisco, San Francisco, CA, United States; ^13^School of Medicine, Vanderbilt University & Vanderbilt University Medical Center, Nashville, TN, United States; ^14^School of Medicine and Richard M. Fairbanks School of Public Health, Indiana University, Indianapolis, IN, United States; ^15^Faculty of Arts and Sciences, Harvard University, Cambridge, MA, United States

**Keywords:** reporting guidelines, music, music therapy, intervention, reporting quality

## Abstract

**Background:**

Detailed intervention reporting is essential to interpretation, replication, and eventual translation of Music-based Interventions (MBIs) into practice. Despite availability of *Reporting Guidelines for Music-based Interventions* (RG-MBI, published 2011), multiple reviews reveal sustained problems with reporting quality and consistency. To address this, we convened an interdisciplinary expert panel to update and improve the utility and validity of the existing guidelines using a rigorous Delphi approach. The resulting updated checklist includes 12-items across eight areas considered essential to ensure transparent reporting of MBIs.

**Objective:**

The purpose of this explanation and elaboration document is to facilitate consistent understanding, use, and dissemination of the revised RG-MBI.

**Methods:**

Members of the interdisciplinary expert panel collaborated to create the resulting guidance statement.

**Results:**

This guidance statement offers: (1) the scope and intended use of the RG-MBI, (2) an explanation for each checklist item, with examples from published studies, and (3) two published studies with annotations indicating where the authors reported each checklist item.

**Conclusion:**

Broader uptake of the RG-MBIs by study authors, editors, and peer reviewers will lead to better reporting of MBI trials, and in turn facilitate greater replication of research, improve cross-study comparisons and meta-analyses, and increase implementation of findings.

## Introduction

Music-based Interventions (MBIs) can be broadly defined as the use of music or music-based experiences to address any dimension of health or human development. Interest in MBIs has grown markedly in the last decade. As with all interventions, detailed reporting of the intervention and its theoretical foundations is essential to interpretation, replication, and eventual translation into practice. However, multiple reviews reveal sustained problems with MBI reporting quality and consistency (Bradt et al., 2021; De Witte et al., 2022; De Witte et al., 2020; Düzgün and Özer, 2020; Yangöz and Özer, 2022; Gao et al., 2019; Jespersen et al., 2022; Monsalve-Duarte et al., 2022; Moreno-Morales et al., 2020; Nguyen et al., 2022; Wang et al., 2021; Wang et al., 2018; Yang et al., 2021; Lepping et al., 2024). MBIs are often challenging to describe due to the complexity of music stimuli, variety of music experiences, and other factors unique to these interventions; however, clear reporting is imperative for the development and advancement of the field. In 2011, Robb and colleagues developed and published *Reporting Guidelines for Music-based Interventions* (Robb et al., 2011). These MBI guidelines focused on detailed reporting of music interventions and were designed to be used in conjunction with methodological checklists, such as the Consolidated Standards for Reporting Trials (CONSORT) (Schulz et al., 2010) and Transparent Reporting of Evaluations with Non-randomized Designs (TREND; Des Jarlais et al., 2004) statements. The 2011 guidelines and checklist are publicly available as an open access article in *Journal of Health Psychology* (Robb et al., 2011) and the EQUATOR Network (n.d.).

Since publication of the original guidelines, there has been exponential growth in the number of published studies investigating the use of music to improve health outcomes (Golden et al., 2021; McCrary et al., 2022; Robb et al., 2018). This increase was accompanied by several landmark events and publications, including the launch of the National Institutes of Health (NIH) *Sound Health Initiative* (National Institutes of Health, 2023) and the National Endowment for the Arts Research Labs (n.d.) that have provided an unprecedented and sustained level of national attention and funding to accelerate music health research (Gordon and Lense, 2020); publication of the *NIH MusicBased Intervention Toolkit* (Edwards et al., 2023; National Institutes of Health, 2023); and the World Health Organization’s first-ever report synthesizing global evidence on the role of the arts to improve health and well-being (Fancourt and Finn, 2019).

Despite calls for improved reporting of scientific studies and availability of the 2011 reporting guidelines, a 2018 review found that overall reporting quality of published MBI reports remained poor (Robb et al., 2018). Less than 50 percent of the reviewed studies offered essential information about the underlying theory (or scientific premise), interventionist qualifications, treatment fidelity, music, or setting. Findings from this and other recent reviews suggest limited use of the guidelines by study authors, reviewers, and journal editors. This may be due to limited awareness, low perceptions of relevance, lack of item clarity, and/or the absence of a published guidance statement to support usage.

To address these concerns, Robb and colleagues convened an interdisciplinary group of experts to update and improve the utility and validity of the current MBI reporting guidelines through a rigorous process that involved: (1) a field scan, (2) a consensus process using Delphi surveys and an interdisciplinary expert panel, and (3) updated *Reporting Guidelines for Music-based Interventions* (RG-MBI; Robb et al., 2025). The updated RG-MBI checklist includes 12 items across eight areas that are considered essential to ensure transparent reporting (Table 1). Readers are referred to the validation study for more details of the methodology used to arrive at the final checklist (see Robb et al., 2025).

**Table 1:** Reporting Guidelines for Music-based Interventions Checklist*.

Item number		Item	Location** (page or appendix number)
1		**Brief Name**† Provide the name or phrase that describes the intervention.	
2		**Intervention Theory and/or Scientific Rationale**Provide a rationale for the music and/or music experience(s). Specify how essential features of the music and music experience(s) are expected to influence targeted outcomes.	
3		**Intervention Content** For Items 3a – 3e, describe the music intervention with enough detail to support replication. When applicable, describe procedures for tailoring the intervention.	
	3a	**Music Selection** Describe the process for how music was selected including who was involved in music selection.	
	3b	**Music** Specify key details about the music that may be relevant to specified outcomes of interest. Characteristics may include compositional features of the music (such as tempo, harmony, rhythm, pitch, tonality, form, instrumentation)‡, sound intensity or volume, lyrics, and/or how the music relates to the participants’ cultural identity and heritage. When using published music, provide reference for a sound recording or sheet music.	
	3c	**Music Delivery Method** Provide details about how music was provided to or created with participants (such as live, recorded, computer generated).‡ Include any details necessary for replication. This might include size of performing group, use of playback equipment, person controlling volume.	
	3d	**Materials** List all materials necessary for the music experience. Include music and non-music equipment and materials.	
	3e	**Intervention Strategies** Describe the music intervention strategy or strategies being studied (such as music listening, improvisation, song writing, rhythmic auditory stimulation).‡	
4		**Interventionist** Specify interventionist qualifications, credentials, training, and/or experience. Indicate how many interventionists delivered the music experience.	
5		**Individual or Group Intervention** Specify whether interventions were delivered to individuals or groups of individuals. For group interventions, specify the size of the group.	
6		**Setting** Describe where the intervention was delivered. Include location, privacy level, ambient sound, and/or any other factors that may have affected participants’ experiences.	
7		**Intervention Delivery Schedule** Report number of sessions, session length (for example, 60 min), frequency (for example, 3x/week), time interval between sessions (for example, single day, three consecutive days), and duration (for example, over 4 weeks).‡ Include practice, experiences, or tasks that are assigned to participants between intervention sessions.	
8		**Treatment Fidelity** Describe strategies and/or measures used to ensure that the music intervention was delivered and received as intended.	

*Reproduced from Robb et al. (2025). The focus of the RG-MBI is on reporting details of the music-based intervention under investigation. Importantly, the checklist was designed to be used in conjunction with methodological checklists such as CONSORT (for randomized controlled trials), SPIRIT for clinical trial protocols, and other study designs (see www.equator-netowrk.org). For example, when reporting findings from a randomized controlled trial, the RGMBI checklist can serve as an extension of Item 5: Interventions on the CONSORT 2010 checklist. **Use N/A if an item is not applicable for the intervention being described. †Item 1 is taken from the TIDieR checklist. Following RGMBI item validation, we ordered RGMBI Items 2–8 to coincide with the order of TIDieR items based on content. ‡Parenthetical details are examples only; they are not intended to be exhaustive.

### Purpose of the guidance statement

The purpose of this explanation and elaboration (E&E) document is to facilitate understanding, use, and dissemination of the RG-MBI to improve reporting quality of music based interventions in published research. To that end, this document elucidates the scope and intended use of the RG-MBI; it then provides further detail for each checklist item, along with examples of optimal reporting, and annotations of published examples.

### Scope and intended use of the RG-MBI

The purpose of the RG-MBI is to assist authors in describing MBIs with sufficient detail to support interpretation and replication. As noted above, MBIs can be challenging to report because they are not only complex and diverse in their content, but also offered in a variety of clinical, virtual, and community-based settings, and provided by a range of professionals— including (but not limited to) credentialed music therapists, healthcare professionals such as nurses and physicians, educators, psychologists, musicians and music practitioners. Given this complexity, the RG-MBI are intended to support authors in determining what to report in their publications for optimal quality and reproducibility; as such, they reflect the most crucial aspects necessary to support replication and evidence synthesis. The guidelines are intended as a necessary minimum starting point, and authors should provide any additional information (beyond the RG-MBI) that they consider necessary for replication and cross-study comparisons. In some cases, a checklist item may not be applicable and as a result would not be reported. The RG-MBI can also be used by authors conducting systematic reviews, and by journal reviewers and editors to assess reporting quality of manuscript submissions.

#### Use with other guidelines

The RG-MBI offers specific guidance on what aspects of a music-based intervention authors should describe in their published research reports. The focus is on the description of the intervention, including its conceptualization and delivery, and not the general methodological approach. The RG-MBI was developed to overcome limitations of reporting guidelines like CONSORT (Schulz et al., 2010) and TREND (Des Jarlais et al., 2004) that provide excellent methodological guidance, but limited direction for reporting complex interventions (Perera et al., 2007; Boutron et al., 2008b; Boutron et al., 2008a; Dijkers et al., 2002). As such, the RG-MBI is intended to be used in conjunction with method-specific guidelines such as CONSORT (Schulz et al., 2010), TREND (Des Jarlais et al., 2004), or SPIRIT (Standard Protocol Items: Recommendations for Interventional Trials; Chan et al., 2013).

#### Order of checklist items

The order of RG-MBI checklist items mirror the TIDieR (Template for Intervention Description and Replication) checklist (Hoffmann et al., 2014) and, as with the TIDierR checklist, it is not meant to indicate the order or priority with which authors report information. In fact, as noted by the authors of TIDieR, it is often possible to combine a number of items from the checklist into one sentence (see annotated article; Supplemental Material, Appendix 1).

#### Compatibility with journal length restrictions

We recognize that journal limits for article length and tables pose challenges with complete reporting. When full reporting is not possible due to publishing constraints, we recommend that authors consider using Supplementary material, or reference a published protocol (or curated open-science website in which Supplementary material is permanently archived), in order to fully report intervention details (see Supplemental Material, Appendix 1). Supplementary materials may include such information as full music 156 transcriptions, listings of audio/video files, or additional detailed descriptive text.

### The RG-MBI checklist explanation and elaboration

RG-MBI Checklist items are shown in Table 1. A downloadable version of the checklist is also available on the EQUATOR Network website. In this section, we provide an explanation for each item, along with examples of reporting from published research. In addition, we provide a published study with annotations indicating where the authors reported each checklist item (Supplemental Material, Appendix 1). These annotations augment the item-specific text-box examples by demonstrating how RG-MBI checklist items can be captured in published reports, including variations in their order and location. While we have strived to incorporate a variety of Music-based Interventions in the included examples, this document is neither exhaustive nor fully representative of all potential MBI types.

### Item 1: Brief name

Provide the name or phrase that describes the intervention.

BOX 1 **Item 1 examples**

1a. Rhythmic Auditory Stimulation (Thaut et al., 1996).

1b. Community Health Intervention through Musical Engagement (CHIME) (Sanfilippo et al., 2020).

1c. Remini-Sing is a once weekly 2-h therapeutic group singing program co-facilitated by two trained music therapists (Tamplin et al., 2018).

Explanation: This item is taken directly from the TIDieR checklist (Hoffmann et al., 2014). Using a name or phrase the succinctly describes the intervention helps readers to quickly identify the type of intervention and facilitates the search for other published reports of the same intervention. We recommend that authors provide the intervention name (Box 1, examples 1a and 1b), explain any abbreviations or acronyms (Box 1, example 1b), or provide a short (one or two line) descriptive statement of the intervention (Box 1, example 1c). Some MBIs, such as Guided Imagery and Music (McKinney and Honig, 2017; Jerling and Heyns, 2020), Rhythmic Auditory Stimulation (Ye et al., 2022; Zhang et al., 2022), or Improvisational Music Therapy (MacDonald and Wilson, 2014), have an established definition and substantive body of published research, whereas other MBIs and their labels are less established. In these situations, we recommend providing a short name and brief description that uses well defined terms.

### Item 2: Intervention theory and/or scientific rationale

Provide a rationale for the music and/or music experience(s). Specify how essential features of the music and music experience(s) are expected to influence targeted outcomes.

BOX 2 **Item 2 examples**

2a. “…in this conceptual framework, music is conceptualized as facilitator of parent coaching. More specifically, music is hypothesized to optimize the psychophysiological arousal of both child and parent, improving the synchronization of their social communication” (Hernandez-Ruiz, 2020, p. 195–196).

2b. “Grounded in self-determination and motivational coping theory (Ryan and Deci, 2017), the CSM-MT [Contextual Support Model of Music Therapy] specifies how music can be used to create supportive environments that encourage learning and enactment of active coping strategies to manage distress (Robb, 2000). Supportive environments offer structure, autonomy support, and relationship support and these principles guided AME [Active Music Engagement] design and tailored delivery” (Robb et al., 2023b, p. 9).

2c. “This pilot study was guided by the theory of ‘Balance between Analgesia and Side Effects’ (Good and Moore, 1996). This widely used theory includes the concept of multimodal interventions, operationalized as pharmacological and nonpharmacological interventions (music listening) to promote optimal pain relief (Good and Moore, 1996). This theory posits that multi-modal pain management will maximize pain relief and reduce side effects” (Laframboise-Otto et al., 2021, p. 87).

Explanation: There needs to be a clear scientific premise for why and for whom an investigator expects the specified use of music to influence the outcome of interest. Including a theory or scientific rationale helps readers identify essential components of the intervention and better understand the mechanisms of action. Theoretically driven approaches also increase the clinical utility of MBIs by clarifying what processes to target and who might derive the most benefit (Morrow et al., 2022).

The rationale can include established theories and/or scientific evidence about social, psychological, perceptual, and/or neurobiological responses to music that support the relationship between components of the music intervention, the hypothesized mechanisms of action, and the intended outcome. In addition to using text (Box 2, examples 2a-2c), diagrams or figures can also be used to illustrate the theoretical model, including potential paths of action between intervention, mediators, and outcomes (see Howlin and Rooney, 2021).

### Item 3: Intervention content

For Items 3a-3e, describe the music intervention with enough detail to support replication. When applicable, describe procedures for tailoring the intervention.

#### Item 3a: Music selection

Describe the process for how music was selected including who was involved in music selection.

BOX 3 **Item 3a examples**

3a.1 “The playlist consisted of 80 predefined different tracks (Supplementary Figure S2). A classical repertoire structured to avoid significant adrenergic stimulation and a raise of cortisol levels was chosen[…] The tempo/rhythm was setup in a range between 60 and 80 beats per minute (bpm), because this range mirrors the human heart rate (HR) and facilitates relaxation” (Burrai et al., 2020, p. 542).

3a.2 “In the favorite music condition, participants listened to their self-selected favorite songs sent to study staff prior to visit. When asked to provide songs for the session, study staff told participants, ‘These [songs] do not have to be anything specific other than songs you enjoy listening to.’ We requested that participants select 7 songs to allow sufficient time to cover the QST [quantitative sensory training] session” (Colebaugh et al., 2023, p. 1184).

3a.3. “A research assistant compiled the music playlists by examining the Billboard charts for popular recordings. The major function of the music within the music listening protocol was to provide comfort, relaxation, and/or distraction” (Burns et al., 2018, p. 90).

3a.4. “The music therapist selected the music for the music and imagery sessions, which consisted of pieces from the Western Art Music and new age genres (Meadows et al., 2015). The music served a number of functions in these sessions […] Thus, music therapists chose music from a list based on an assessment of the patient’s need for structure and energy level (Meadows et al., 2015)” (Burns et al., 2018, p. 89).

Explanation: In addition to describing the music used (checklist Item 3b), authors should also describe the process for *how* music was selected and *who* was involved in selecting the music. There are many approaches to music selection, including selection by the researcher (Box 3, example 3a.1; 3a.3), the study participant (Box 3, example 3a.2), through therapist assessment (Box 3, 3a.4), or the use of computer-generated playlists or algorithms. The theory and/or scientific rationale, reported in checklist Item 2, will inform how and by whom music is selected. In some studies, compositional or psychoacoustic features may be central to music selection (Box 3, example 3a.1 and 3a.4). For other studies, music selection might be based on familiarity, genre, cultural relevance, or participant preference (Box 3, example 3a.2).

#### Item 3b: Music

Specify key details about the music that may be relevant to specified outcomes of interest. Characteristics may include compositional features of the music (such as tempo, harmony, rhythm, pitch, tonality, form, instrumentation), sound intensity or volume, lyrics, and/or how the music relates to the participants’ cultural identity and heritage. When using published music, provide reference for a sound recording or sheet music.

BOX 4 **Item 3b examples**

3b.1. “Frequency modulation and music pieces used in this arm are comparable to the procedures of applications of AVWF [Audiovisuelle Wahrnehmungsförderung] -based music interventions in previous clinical studies (Olbrich et al., 2015; Olbrich and Näher, 2017) […] Six different mixes of music pieces are chosen that cover a wide range of genres such as classic, instrumental, pop, rock, and world music (see Box 2 for details). Three of these mixes are compositions of known artists of which two also contain vocals. The remaining three of the music mixes are instrumental music pieces […] According to the AVWF method, the applied music is modulated within the audible frequency range of 50–4,000 Hz via a software system. This involves filtering the harmonic overtones of low frequencies of the music pieces presented in music-listening sessions 1–7. In sessions 8–10, modulation will be additionally applied to frequencies in the high spectrum” (Feneberg et al., 2020, p. 9).

3b.2. “The manual describes a certain degree of flexibility to adapt to the needs of the individual from moment to moment. The music therapist used the music therapy synchronization technique (Aalbers et al., 2019; Bruscia, 1987), verbal reflection and ER-card to address five components of ER (emotion regulation). […] The synchronization technique was used as a mirroring technique, performing what the student does simultaneously, timing so that their actions tend to synchronize without the interference of talking (Bruscia, 1987). The music therapist used musical components in line with the client’s musical improvisation, e.g., rhythm or melody, to synchronise the client’s musical improvisation and to evoke changes in ER” (Aalbers et al., 2022, p. 135).

3b.3. “Music was sedative, 60–80 beats per minute without lyrics, with a sustained melodic quality, and with controlled volume and pitch (Taylor, 1973; Zeller et al., 1997). There were four audiotapes, two of Taiwanese music and two of American music. Taiwanese folk songs and Buddhist music were selected by the Taiwanese investigator (S.T.H.), while harp music and piano music were selected by an American investigator” (Huang et al., 2010, p. 1356).

3b.4. “Sound levels were measured using a decibel meter (BAFX Products^®^, Muskego, WI) and maintained below the recommended AAP standard of 45dBA with the max transient volume of 60 dBA (1-s LMax)” (Anbalagan et al., 2024, p. 680).

Explanation: Authors should provide a clear description of the music provided and/or created during the intervention, providing details about the features of music that are (a) thought to be important to the intervention and that (b) correspond with the intervention theory and/or scientific rationale specified for checklist Item 2.

The theoretical or scientific framework used by a given study or intervention to guide music selection can inform authors’ decisions about what information to include in their published report. For example, in their study comparing the differential effects of frequencymodulated and unmodulated music on experimentally induced pain, Feneberg et al. (2020) reported features of the music expected to modulate Autonomic Nervous System (ANS) activity, with additional details about genre, instrumentation, and the presence/absence of vocals (Box 4, example 3b.1). The authors also included a table listing song selection titles and recording artists (Feneberg et al., 2020).

We recognize that, for MBIs in which the interventionist and/or participant are *creating* the music (e.g., improvisational or songwriting interventions), the music cannot be described *a priori*. In these situations, the author might describe parameters for how the investigator facilitated and structured the music-making experience. For example, when Aalbers et al. (2022) reported on their manualized improvisational music therapy program, they described each theoretically derived component of the intervention in detail, along with the corresponding techniques used to facilitate the improvised music experience with participants (Box 4, example 3b.2 offers an excerpt). For additional examples, see Santos et al. (2020) and Haslbeck and Bassler (2020), which also includes video examples as Supplementary material.

As mentioned earlier, in addition to providing descriptive information in the main text, authors can offer more detailed information about the music as Supplementary material. This might include music transcriptions, song titles or playlists, references for published music arrangements, music recordings/artists, and/or audio-recordings of improvised or newly composed music. Use of Supplementary material allows authors to provide greater detail about key features of the music that are known (or hypothesized) to have *direct* relevance to the outcome of interest, as well as other features of the music that may prove to be an important modulator of primary intervention effects in future or related studies. For example, using music that is relevant to one’s cultural heritage has the potential to modulate the effectiveness of the intervention (Box 4, example 3b.3). Reporting on volume (measured in decibels) can also be important to report (Box 4, example 3b.4), given that noise levels may affect participant safety and outcomes (Marik et al., 2012; Etzel and Balk, 1997).

Note that reporting for Item 3b need not be limited to music-theoretic terms; it can include music informatics measures (e.g., Lartillot et al., 2008) or other music analyses. For example, in a study of the effects of tempo on movement amplitude, merely specifying the music tempo may be inadequate, as other aspects of music—such as ‘event density’ (how ‘busy’ the music is), ‘pulse clarity’ (how clearly the tempo pulse is conveyed), degree of syncopation, and overall energy—could also influence the outcome.

#### Item 3c: Music delivery method

Provide details about how music was provided to or created with participants (such as live, recorded, computer generated). Include any details necessary for replication. This might include size of performing group, use of playback equipment, or person controlling volume.

BOX 5 **Items 3c examples**

3c.1 “The participants listened to a rhythmic pattern played live by the music therapist and when they wished, they created and combined musical patterns with instruments, voices, or bodies, spontaneously creating music according to the context provided by the base pattern” (Diaz Abrahan et al., 2023, p. 52).

3c.2. “The musical stimuli were presented as a live orchestral performance and the concert was programmed as part of the BBC 3 Free Thinking Festival. … [Listed compositions] were played, in full, by the Royal Northern Sinfonia” (O’Neill and Egermann, 2022, p. 6).

3c.3. “Participants were invited to share two of their significant songs with the group. One song was to be sung individually with harmonic accompaniment and support from the therapist, and the other song was played to the rest of the group through a high-quality loudspeaker” (Pérez-Aguado et al., 2024, p. 128).

3c.4. “Music in the experimental group was…administered through an iPod dock. All participants listened to the same recordings via ambient speakers…The volume of the music was set in advance and was the same for each child” (Hartling et al., 2013, p. 828).

Explanation: While most authors report whether an MBI was delivered using live or recorded music, many leave out more detailed delivery information (Robb et al., 2018). Note that this item’s emphasis is on the *delivery method*; not on characteristics of the music (checklist Item 3b) or on materials used (checklist Item 3d).

When using *live music*, we recommend that authors report who created (or performed) the music. For example, music might be created by the person delivering the intervention (Box 5, example 3c.1), by a performer (Box 5, example 3c.2), by the interventionist together with study participant(s) (Box 5, example 3c.3), or by the participant alone. In addition, details such as the number of performers, audience size, and other relevant social dynamics (Box 5, example 3.c.2) may be valuable.

When using *recorded music*, computer-generated music, or virtual platforms, we recommend that authors specify how the music was delivered, including specific information about the equipment used to deliver recorded music (Box 5, example 3c.4 and 3c.5). The use of speakers, headphones, or virtual platforms each creates a different listening experience. For example, some studies have elected to use headphones (Bluetooth hearing aids; earbuds; speaker pillows) to deliver music while reducing external, environmental sounds (for examples see Feneberg et al., 2020 and Aravena et al., 2020). Other studies have delivered music using speakers to facilitate shared (or group) music listening, with the aim of enhancing social bonding and cohesion (Box 5, example 3c.3).

With recorded music, investigators should also report on who controlled music playback and volume (Box 5, example 3c.4). For example, volume may in some cases be monitored/controlled by investigators to protect recipients’ hearing – especially when sound levels might affect neurological development (e.g., premature infants) or when participants lack the ability to selfmonitor (e.g., post-operative recovery; patients in a coma). Similarly, some studies may require that playback be facilitated by the study investigator, while in other studies, participants control playback and/or volume for themselves.

Finally, telehealth platforms are increasingly being used to deliver music interventions, due in part to transitions made during the COVID 19 pandemic to deliver MBIs to recipients isolated at home (Knott and Block, 2020). Given that the quality of telehealth music delivery can be affected by the equipment (e.g., microphones, speakers) and choice of platform (e.g., Zoom, WebEx, Teams, Doxy; Story et al., 2024) reporting these details supports the evaluation and comparison of telehealth vs. in-person music delivery.

#### Item 3d: Materials

List all materials necessary for the music experience. Include music and non-music equipment and materials.

BOX 6 **Item 3d examples**

3d.1 “Materials used consisted of, but were not limited to, a six-string acoustic steel string guitar, hand-held percussion instruments such as tambourines, paddle drums, egg shakers, and electronic instruments accessed through GarageBand on an iPad” (Rushing et al., 2021, p. 44).

3d.2 “Fine motor exercises were performed by participants using touchscreen instruments on iPads, with Garageband™ and Thumbjam™ software installed, and using a touchscreen plectrum requiring pinch-grip. Acoustic instruments on stands and some hand percussion were used for proximal, gross movement” (Street et al., 2019, p. 384).

3d.3 “Each patient received Beats ™ Solo 3 Wireless headphones (Copyright ™ 2017 Apple Inc. All rights reserved) with active noise control connected to a Bluetooth-enabled audio player” (Aravena et al., 2020, p. 3).

Explanation: Authors should describe all materials (music and non-music) relevant to intervention delivery with enough detail to support study replication. Materials will vary depending on the type of intervention. For example, in an active music-making intervention, music materials may include specific musical instruments, computer programs, or devices used to create music. Materials may also include other non-music materials like lyric sheets, sheet music, or props such as toys to companion a young child’s music making experience or scarves to encourage expressive movement while singing (Box 6, examples 3d.1 and 3d.2). For other types of interventions, it may be important to specify equipment such as speakers, headphones, earbuds, or speaker pillows (Box 6, example 3d.3).

In cases where sound quality might affect a participants’ experience, we recommend authors provide details about the make or model of the musical instruments, technology, or playback equipment used (Box 6, example 3d.3). The description of materials becomes even more useful when authors include their decision-making process for materials selection, including aspects of materials considered essential (or less relevant) to replication, and why. We recommend that authors provide detailed information about materials in their description of the intervention, in tables, or as Supplementary material.

#### Item 3e: Intervention strategies

Describe the music intervention strategy or strategies being studied.

BOX 7 **Item 3e examples**

3e.1 *“*The Melomics Health group listened at home, for 2 weeks, to a daily 30-min-playlist created by an algorithm with a ‘therapeutic’ logic. Melomics Health music aims at improving clinical conditions and reducing symptoms. […] Melodies are composed on the basis of experimenter-selected music parameters like timbre, tonal environment, tempo, intervals, pitches, dynamics and density, according to the therapeutic aims of relaxing and deactivation. Knowledge from biomedicine, neurosciences, music psychology and clinical practice inform and support these choices, on the basis of which Melomics-Health composes the musical works (for an example see Supplementary material in Raglio and Vico, 2017).” (Raglio et al. 2020, p. e84).

3e.2 The group singing intervention was designed with the goal of promoting psychosocial well-being among adults. Each session included activities that targeted the hypothesized pathways by which group singing could promote psychosocial well-being. These pathways are highlighted in our conceptual model. To provide psychosocial engagement, the sessions included working toward a common goal (e.g., learning new songs and choreography/movement to the songs, working toward a performance), activities to promote group cohesion (e.g., group vocal warm-ups, discussion of the meaning and cultural traditions of the songs, sharing personal memories associated with the songs), emotion regulation (e.g., discussing the emotions elicited by the music and group singing), and socialization (e.g., a 10 min break for refreshments) (Johnson et al., 2020).

Explanation: MBIs might have a single-component or a multi-component strategy. In singlecomponent MBIs, a single, specific component is used, such as listening to recorded music (Box 7, example 3e.1). The majority of MBIs, however, are multi-component (Robb et al., 2018), making it essential that authors report all component strategies that comprised the intervention (Box 7, example 3e.2). For example, a “songwriting intervention” could involve composing original music and lyrics, writing original lyrics using the chord sequence of an existing song, and/or replacing partial lyrics of an existing song. In addition, a songwriting intervention may include various brainstorming activities, journaling between sessions, or other exercises that can influence or enhance the intervention and its outcomes. For this item, we recommend that authors use text and/or tables to define and describe the intervention. In addition, we recommend that authors identify the relationship of the intervention strategies to the theoretical/scientific rationale specified for checklist Item 2.

### Item 4: Interventionist

Specify interventionist qualifications, credentials, training, and/or experience. Indicate how many interventionists delivered the music experience.

BOX 8 **Item 4 examples**

4a. “Intervention was conducted by 11 NICU-specialized music therapists with master’s degrees in MT [music therapy] (or in terminal stage of degree [2 therapists]) who received training and supervision in the study intervention” (Ghetti et al., 2023, p. 3).

4b. “[…] two experienced physiotherapists provided the intervention, both with a bachelor’s degree and certified RGM [Ronnie Gardiner Method] practitioners […] Both practitioners had several years of practice from teaching RGM (4 and 10 years, respectively) and of working with people with PD [Parkinson’s Disease], individually as well as in groups (27 and 19 years). The interventionists both had much experience of creating choreoscores suitable for different neurological diseases and age groups” (Pohl et al., 2020, supplemental file ii, p. 2).

4c. “The music therapist who delivered the intervention was a Fellow of the Association for MI [Music and Imagery] (the designation following completion of advanced GIM [Guided Imagery and Music] training)” (Story et al., 2024, p. 296).

Explanation: The term “interventionist” refers to the person (or persons) who provided the music intervention. MBIs are delivered by a variety of professionals with varied levels of experience and training in music and/or the therapeutic application of music (Golden et al., 2021; Robb et al., 2018). We recommend authors report the *qualifications* of the interventionist(s), including professional background, credentials, and training, such as degrees and certifications that highlight relevant expertise (Box 8, examples 4a-4b). Also, when possible, note past practical experiences that align with the intervention’s context, demographic, or modality (Box 8, example 4b). These details provide important information about the pre-existing skills, expertise, and experience that may influence or be essential to the intervention, and they have implications for the translation of research into practice, cross-study comparisons, and research about the level of expertise required to effectively deliver an MBI.

It is also important to report the *number* of interventionists involved in delivering the intervention, as this can significantly influence implementation and outcomes. For instance, an intervention led by a single interventionist (Box 8, example 4c) may entail a different approach compared to one conducted by a multidisciplinary team (see Gonzalez-Hoelling et al., 2021). Specifying the number of interventionists also allows for consideration of intervention-versus person-effects (Box 8, example 4a).

Finally, if applicable, report the *relationship* the interventionist(s) had with participants at the time of the study. Relationships have been shown to influence outcomes in some cases; for example, studies that involve participants who are already familiar with the interventionist might yield different effects compared with studies in which the interventionist is unknown to the participant at the time of study enrollment.

### Item 5: Individual or group intervention

Specify whether interventions were delivered to individuals or groups of individuals. For group interventions, specify the size of the group.

BOX 9 **Item 5 examples**

5a. “The participants of the intervention group (music therapy) receive 30 min of *individual music therapy* twice a week for 12 weeks in their own room” (Baroni Caramel et al., 2024, p. 4).

5b. “Each child–parent dyad attended ten 18 − min experimental sessions” (Samadani et al., 2021, p. 3).

5c. “The treatment group will receive social skill intervention using music therapy in groups of eight” (Yum et al., 2020, p. 4).

Explanation: We recommend authors indicate whether the intervention was delivered to one person at a time (Box 9, example 5a), to one dyad at a time (Box 9, example 5b), or to a group – including the number of people in the group (Box 9, example 5c). For group interventions, we also recommend that authors indicate whether group composition was consistent (i.e., same individuals repeatedly attend same group) or varied (see Dahms et al., 2021).

The number of people experiencing an intervention can affect intervention delivery, receipt, underlying processes, and outcomes (Good and Russo, 2022; Guo et al., 2021; Osborn et al., 2006; Tachibana et al., 2018; Wheeler et al., 2003). Group interventions may, for example, provide added value through peer support, accountability to peers, and social relationships; whereas an individual approach offers increased contact with the interventionist and can support tailored delivery of intervention content based on individual needs. The unit of delivery also has cost implications that can affect scalability, with effective group-based interventions increasing capacity while also reducing cost (Chlan et al., 2018; Dickson et al., 2009; Gaviola et al., 2024).

### Item 6: Setting

Describe where the intervention was delivered. Include location, privacy level, ambient sound, and/or any other factors that may have affected participants’ experiences.

BOX 10 **Item 6 examples**

6a. “The study will be conducted in a 24-bed, level III, tertiary NICU, certified in providing care according to the NIDCAP model. The unit is constructed of three, open-space rooms. Each infant has his/her own facilities with a space for parents, as well as for care-givers. A decorative curtain can be placed around the space for privacy. The first and second sessions (i.e., during hospitalization) will occur in the open-space NICU. The three-month follow-up session will usually take place in the family’s home, but may be held at the hospital, outside of the NICU, according to the parent’s choice” (Yakobson et al., 2020, p. 225).

6b. “The PMI [psychotherapy with music intervention] was delivered at the Psychiatry Institute of the “Maggiore della Carita” University Hospital, Novara, Italy. A large room was used to host the group, where participants and therapists could sit in a circle. The environment was quiet and granted a proper privacy level” (Zeppegno et al., 2021, p. 4).

6c. “Patients were asked to walk alone outside in a safe environment (with no cars, without crossing roads, and on regular ground) while listening to musical stimuli for 30 min, five times a week, for 4 weeks. During each session, they could stop up to four times and for maximum of combined 10 min” (Cochen De Cock et al., 2021, p. 2).

6d. “…participants will use their personal device (computer, tablet, phone) to connect to the virtual sessions in a quiet, private setting of their choice” (Story et al., 2023).

Explanation: Qualities of the environment can influence participants’ engagement in and effectiveness of an intervention. We recommend that authors provide a detailed description of the setting where the music-based intervention took place, including general location, privacy, ambient sound, and any other factors that might affect replication. The general location (e.g., clinic, school, therapist’s office, home) is important, as are the aesthetics and finer elements of each setting (Box 10, example 6a). If there are multiple settings, describe each setting. If the MBI was provided virtually or through telehealth, the setting may vary depending on the home or work environment of the provider and recipient. In these cases, we recommend authors report setting related information specified in their intervention protocol (Box 10, example 6d).

In addition to general location, privacy levels and ambient sound may be important to report. Depending on the intervention, delivery in a private space versus a more communal area may produce different outcomes due to potential effects on participants’ comfort, ability to focus, and willingness to engage (Box 10, example 6a). Because MBIs involve auditory stimuli, additional sounds in the environment can also affect study participation and outcomes. For example, an intervention delivered in a private space with minimal ambient sound may yield different findings than the same intervention delivered in an open space where sounds from televisions, family/staff conversations, and other activities are audible (Box 10, example 6b). Examples of additional factors that might affect MBI implementation and outcomes include accessibility, size or layout of the room, lighting conditions, or aspects of the environment relevant to participation (Box 10, example 6c).

### Item 7: intervention delivery schedule

Report number of sessions, session length (for example, 60 min), frequency (for example, 3x/week), time interval between sessions (for example, single day, three consecutive days), and duration (for example, over 4 weeks). Include practice, experiences, or tasks that are assigned to participants between intervention sessions.

**BOX 11 Item 7 examples**

7a. “The experimental group met every Monday for four consecutive weeks and lasted approximately 50 min per session” (Suh, 2023, p. 685).

7b. “Infants and parents in the NICU MT group received 3 individual MT sessions per week throughout hospitalization, with maximum 27 sessions, lasting approximately 30 min each (with time in active music making depending upon infant tolerance)” (Ghetti et al., 2023, p. 3).

7c. “Since previous research indicates that chronobiological rhythms influence perceived pain and stress parameters (Sammito et al. 2016; Hagenauer et al., 2017) the appointments will be scheduled exclusively between 12 and 6 p.m. The 10 music-listening sessions (intervention period) will be scheduled within 3 consecutive weeks. Baseline and post-assessments will be held as closely in time as possible to the first and last music-listening session, respectively. Some degree of variability between participants will be accepted in order to better enable participants to fit the large number of appointments into their daily schedules” (Feneberg et al., 2020, p. 5–7).

Explanation: The intervention delivery schedule provides important information about participants’ exposure to an intervention, including the: (1) *number* of sessions; (2) *length* of each session (e.g., minutes/h); (3) *frequency* of sessions – how often the exposure happens over a specified period of time (e.g., 3x/week); (4) *time interval between sessions* – when applicable, indicate time between sessions; and (5) *duration* of program over time (e.g., over 4 weeks). Authors may want to specify the duration of time the participant experienced (or was exposed to) music versus other non-music experiences. See Box 11, examples 7a-7c. Intervention delivery details are central to understanding the total dose of or exposure to a behavioral intervention and its potential relationship to outcomes (Voils et al., 2014).

Authors should also indicate whether they used a *fixed* intervention delivery schedule (Box 11, example 7a) or if delivery could be *varied* based on a specified set of rules (Box 11, example 7b) or needs of the participants (Voils et al., 2014). For some studies, timing of the intervention in relation to specific events might also be important to report. For example, the start of the intervention may be contingent on an event, or a specified window of time following a diagnosis (for reporting example see Robb et al., 2023a). Finally, as described in treatment fidelity (Checklist Item 8), the dose of the intervention that participants ultimately receive might differ from the amount intended. For Checklist Item 7, we recommend authors report the delivery schedule (or intended dose) as part of the intervention description, and then report the dose received (part of Checklist Item 8) in the results (Box 12, example 8c).

### Item 8: Treatment fidelity

Describe strategies and/or measures used to ensure that the music intervention was delivered and received as intended.

BOX 12 **Item 8 examples**

8a. “We trained MT-BCs to deliver both intervention and attention control conditions to minimize risk for unmasking evaluators and control for provider differences. All MT-BCs received the same training on standardized protocols and participated in bi-monthly calls. Risk for experimental drift, bias, and diffusion were addressed using self-and external quality assurance monitoring procedures for video recorded sessions” (Robb et al., 2023b, p. 3).

8b. “Ensure that participants understand the information provided in intervention, especially when participants may be cognitively compromised: Videos of sessions reviewed to see whether participants were following the directions of the interventionist. Discussion in supervision sessions with interventionists to improve explanations and demonstrations of activities to participants” (Baker et al., 2019, p. 135).

8c. “Of the 22 participants in the Vocal Music Therapy treatment arm who completed the 8 week program, 19 (86%) attended 7 to 8 sessions, and three (14%) attended 4 to 5 sessions, suggesting that the treatment was well accepted. The main reasons for not attending sessions were (1) transportation issues, (2) weather, (3) doctor appointments, and (4) family emergencies” (Bradt et al., 2016, p. 8).

Explanation: We recommend authors report three aspects of treatment fidelity recommended by the NIH Behavior Change Consortium (Bellg et al., 2004; Borrelli et al., 2005; Borrelli, 2011): (1) *Treatment delivery*: strategies used to ensure the intervention was delivered as intended (Box 12, example 8a), (2) *Treatment receipt*: strategies used to monitor whether participants understood and could demonstrate competence in using or acquiring intervention-related skills (Box 12, example 8b), and (3) *Dose receipt*: whether the intervention dose that participants received differed from what the investigators intended (Box 12, example 8c) (Bellg et al., 2004).

For *treatment delivery*, we recommend that authors describe procedures for training the interventionists and monitoring fidelity for both the intervention and control conditions (Bellg et al., 2004; Nápoles and Stewart, 2018). Procedures may include the use of standardized training materials, an intervention manual, fidelity checklists, monitoring, and/or action plan for retraining when interventionists fall below an operationally defined treatment fidelity score. Training and monitoring help ensure that interventionists deliver study conditions consistently across participants and over time (Box 12, example 8a) (Bellg et al., 2004).

For *treatment receipt,* we recommend that authors describe any strategies they used to assess whether the intervention was received and understood by the participant (Bellg et al., 2004). Example strategies include the use of assessments, active questioning, self-monitoring tools, or role playing (Borrelli et al., 2005). Without monitoring, investigators are unable to determine if null findings are due to an intervention being truly ineffective or due to participants’ limited understanding of or ability to use the skills (Box 12, example 8b) (Bellg et al., 2004).

Finally, for *dose receipt,* we recommend authors indicate whether the intervention dose that participants received was the same as specified in their study protocol. Notice that for Checklist Item 7 we ask authors to report the intended intervention dose. Authors typically report this information in the methods section of their manuscript. Here, for Checklist Item 8, we ask that authors report the dose that participants actually received. Authors typically report this information in the results section of their manuscript (Box 12, example 8c) (Allison et al., 2020).

## Conclusion

The updated RG-MBI checklist and E&E statement are intended to provide guidance to help ensure consistent, transparent reporting of the experimental details and conceptual rationale of Music-based Interventions. Given limited uptake and use of the original RG-MBI checklist, we completed a rigorous consensus process that involved an interdisciplinary group of experts to examine content, item clarity, and utility – working to ensure the checklist’s relevance to the growing community of MBI investigators. Broader uptake of the RG-MBI by authors, editors, and peer reviewers will lead to better reporting of MBI trials, and in turn facilitate greater replication, along with improved cross-study comparisons, systematic reviews, and implementation of findings. We encourage investigators to use the RG-MBI to inform the design of their interventions and dissemination of their work. We also encourage investigators to support increased uptake of the RG-MBI by asking their respective professional organizations to endorse its use. Similarly, we recommend that journal editors and funding agencies endorse the RG-MBI checklist to improve the consistency and quality of research reports, grant submissions, and their review. We anticipate that collective adoption will improve the reporting quality of MBI research and help to accelerate scientific understanding about how music can be used to improve our health, development, and well-being.
