# Supplementary material for: Reporting Guidelines for Music-based Interventions checklist: explanation and elaboration guide
Source: Front Psychol. 2025 Jun 11;16:1552659. doi: 10.3389/fpsyg.2025.1552659 (PMC12188939; doi:10.3389/fpsyg.2025.1552659)
Supplement: Supplementary file 1 [file Data_Sheet_1.pdf]

Appendix 1 Annotated Article.

In this annotated example we highlight where each checklist item is reported. In addition to the annotation in the body of the article, the authors provide further details of the MBI in the supplementary files which are also annotated here.

For the full open-access article, please see the journal website or locate the article using the following citation: Pohl P, Wressle E, Lundin F, Enthoven P, Dizdar N. Group-based music intervention in Parkinson’s disease–findings from a mixed-methods study. Clinical rehabilitation. 2020 Apr;34(4):533-44.

Check for updates

Original Article

CLINICAL REHABILITATION

Item 1. Brief Name

Group-based music intervention in Parkinson's disease – findings from a mixed-methods study

Clinical Rehabilitation
2020, Vol. 34(4) 533–544
© The Author(s) 2020
Article reuse guidelines:
sagepub.com/journals-permissions
DOI: 10.1177/0269215520907669
journals.sagepub.com/home/cre
SAGE

Petra Pohl[1], Ewa Wressle[2], Fredrik Lundin[3], Paul Enthoven[4] and Nil Dizdar[3]

Abstract

Objective: To evaluate a group-based music intervention in patients with Parkinson's disease.
Design: Parallel group randomized controlled trial with qualitative triangulation.
Setting: Neurorehabilitation in primary care.
Subjects: Forty-six patients with Parkinson's disease were randomized into intervention group ($n=26$), which received training with the music-based intervention, and control group ($n=20$) without training.
Interventions: The intervention was delivered twice weekly for 12 weeks.
Main measures: Primary outcome was Timed-Up-and-Go subtracting serial 7's (dual-task ability). Secondary outcomes were cognition, balance, concerns about falling, freezing of gait, and quality of life. All outcomes were evaluated at baseline, post-intervention, and three months post-intervention. Focus groups and individual interviews were conducted with the intervention group and with the delivering physiotherapists.
Results: No between-group differences were observed for dual-task ability. Between-group differences were observed for Falls Efficacy Scale (mean difference (MD) = 6.5 points; 95% confidence interval (CI) = 3.0 to 10.0, $P=0.001$) and for Parkinson Disease Questionnaire-39 items (MD = 8.3; 95% CI = 2.7 to 13.8, $P=0.005$) when compared to the control group post-intervention, but these were not maintained at three months post-intervention. Three themes were derived from the interviews: *Expectations versus Results, Perspectives on Treatment Contents*, and *Key Factors for Success*.
Conclusion: Patient-reported outcomes and interviews suggest that the group-based music intervention adds value to mood, alertness, and quality of life in patients with Parkinson's disease. The study does not support the efficacy in producing immediate or lasting gains in dual-tasking, cognition, balance, or freezing of gait.

[1]Department of Activity and Health, and Department of Health, Medicine, and Caring Sciences, Linköping University, Linköping, Sweden
[2]Department of Acute Internal Medicine and Geriatrics, Department of Social and Welfare Studies, Linköping University, Linköping, Sweden
[3]Department of Neurology, and Department of Clinical and Experimental Medicine, Linköping University, Linköping, Sweden
[4]Pain and Rehabilitation Centre, and Department of Health, Medicine, and Caring Sciences, Linköping University, Linköping, Sweden

Corresponding author:
Petra Pohl, Department of Activity and Health, and Department of Health, Medicine, and Caring Sciences, Linköping University, 581 83 Linköping, Sweden.
Email: petrapohl65@gmail.com

Keywords
Parkinson's disease, randomized controlled trial, mixed-methods, music-based intervention, rehabilitation

Received: 23 July 2019; accepted: 27 January 2020

Introduction

Item 2. Theory/ scientific rationale

Music-based interventions have been suggested as adjunct management options for patients with Parkinson's disease.[1] Dancing, for example, has been shown to improve cognitive dual-tasking, gait-related outcomes, and global cognition.[2] Impaired motor-cognitive dual-tasking is a common deficit in patients with Parkinson's disease,[3] which may be improved with targeted interventions.[4] To increase the attractiveness, musical elements may be incorporated within such interventions,[5] and the social benefits may be further enhanced if the intervention is group-based.[2]

The music-based intervention Ronnie Gardiner Method involves multitasking activities that require the participants to quickly shift attention between motor and cognitive tasks by interpreting visual symbols, synchronizing arms and legs in complex coordinated movements, while simultaneously pronouncing a certain word to the beat of music.[6] Apart from multitasking, the training has the potential to improve bradykinesia, balance, freezing of gait, and cognitive function in patients with Parkinson's disease.[6] The therapy is practitioner-led and usually group-based for the benefits of social experiences and emotional well-being.[6]

Few studies have evaluated the music-based group therapy to date. A randomized controlled trial on stroke survivors found long-term effects on the perception of recovery, balance, grip strength, and working memory compared to controls.[7] With respect to Parkinson's disease, a small feasibility study revealed no between-group differences, but some tendencies towards improved mobility, cognition, and quality of life within the intervention group.[8] A larger study is therefore needed to further investigate possible effects for patients with Parkinson's disease.

When evaluating novel complex interventions, both objective and subjective evaluations should be considered.[9] Including qualitative approaches provides a more in-depth understanding about the intervention. To further broaden the perspectives, the delivering professionals should be included in the evaluation.[9] The aim of this randomized trial was to evaluate the Ronnie Gardiner Method in Parkinson's disease and to gain insights into participants' and therapists' experiences of the group-based music intervention to optimize the contents, delivery, and acceptability and to facilitate further development.

Methods

This was a single-blinded, parallel group randomized controlled trial, integrating data from qualitative methods.[10] The study was approved by the Regional Ethical Review Board of Linköping (Dnr 2016/179-31), and all participants signed an informed consent form after receiving oral and written information. The trial was registered at ClinicalTrials.gov (NCT02999997). The study was conducted following the recommendations of Consolidating Standards for Reporting Clinical Trials (CONSORT)[11] (Supplementary file I) with the extension to the reporting guidelines for music-based interventions[12] (Supplementary file II) and the Consolidated Criteria for Reporting Qualitative data (COREQ).[13]

The following inclusion criteria were used for this study: community-dwelling individuals from 18 years of age with a diagnosis of Parkinson's disease and Hoehn and Yahr[14] up to stage 3, stable medication ⩾four months, and capacity to walk 10 m without gait assistance. To enhance the generalizability of the findings, any medical treatment, even surgical, was accepted. Neurologists screened medical records from the Departments of Neurology and Geriatrics to identify potential study participants of both genders, who were then

contacted by telephone by the first author (P.P.) between December 2016 and August 2017.

Recruited patients underwent a full clinical assessment by specialists in movement disorders at the University hospital and were excluded if they had other neurological deficits or serious health conditions that would compromise participation; significant visual or hearing impairments that would make participation impossible; or severe motor fluctuations. Demographic data included age, gender, disease duration, education level, and fall history the last 12 months. In relation to fall history, patients were asked: ‘Are you experiencing poor balance?’ with a ‘Yes’ or ‘No’ response option. The Unified Parkinson’s Disease Rating Scale[15] was also included.

After the initial examination, included patients were referred to an occupational therapist for cognitive tests, followed by physical tests by the first author. The same assessors performed cognitive and physical re-evaluations within two weeks post-intervention and three months post-intervention. Both assessors remained blind to group allocation at all evaluations.

After baseline assessments, patients were randomized into two groups: intervention group and control group. The randomization procedure was performed by an independent investigator (not part of the study) with numbers generated by a randomization website (www.random.org), and two standardized information letters were sent to the patients depending on group allocation. All patients were asked to refrain from initiating new exercise programmes or other allied health therapy interventions during the study period and were instructed not to share their treatment information to the assessors.

The primary outcome was the Timed-Up-and-Go subtracting serial-7’s measuring the effect of cognitive demands on functional mobility (motor-cognitive dual-tasking). Serial-7’s was chosen instead of the more common serial-3’s subtraction in order to place an even greater demand on the cognitive processes for attention and working memory.[16] Secondary outcomes included (1) cognitive function (Montreal Cognitive Assessment Scale (MoCA);[17] and three parts of the Cognitive Assessment Battery[18] (Test Recall Test (immediate and delayed); Stroop Color-Word Test; and Symbol Digit Modalities Test)) and (2) dynamic balance (Mini-BESTest).[19] Three questionnaires were administered (Falls Efficacy Scale International;[20] Freezing of Gait Questionnaire;[21] and Parkinson Disease Questionnaire 39-items Global Index Score,[22] which rates the quality of life from excellent (zero) to very poor (100)).

Patients were tested while in on-phase, that is, within 1–2 hours after taking their anti-Parkinson medication. Due to practical reasons, it was not possible to re-test patients at the exact same time of day post-intervention. Levodopa equivalent dosage was registered before and after study completion.

Qualitative methodology was used to explore the experiences of the participants and the intervention therapists.[9] To enhance data richness, focus group methodology was combined with individual interviews.[23] In short, focus groups were conducted with patients from the intervention group and with the two delivering therapists by E.W. Additional face-to-face interviews were conducted with eight patients by physiotherapy students. To increase transparency and to ensure dependability and confirmability, an audit trail is provided including theoretical framework, reflexivity, and the process for qualitative analysis (Supplementary file III).

The intervention was delivered in a group setting (14 and 12 participants respectively) at a neurorehabilitation centre twice a week for 12 weeks (60 min/session). Each session was initiated with soft stretching movements and breathing exercises, followed by 50 minutes of exercises typical for the Ronnie Gardiner Method,[6] and ended with winding down to soft classical music. Two physiotherapists, who were not authors, were engaged to provide the intervention; both were certified practitioners of the Ronnie Gardiner Method. Progression of the exercises was determined by the skill of the participants in performing the movements. Intervention details are available in Supplementary file II. A third certified practitioner who was not part of the study performed one integrity check, that is, that the protocol was followed as intended, after six weeks. Homework was given, but not on a regular basis. Training diaries were written to monitor compliance and adverse events.

Item 5. Group or Individual
Item 6. Setting
Item 7. Delivery SChedule

Item 4. Interventionist

Item 3a, 3b, 3c, 3d, 3e

Item 8. Treatment Fidelity

The following annotated document is the supplementary file for Pohl et al., 2020 which includes additional details of the MBI.

Supplementary file II. Checklist for Reporting Music-based Interventions (Robb et al, 2011)

Music-based Intervention Reporting Criteria

This checklist is based on the recommendations by Robb *et al* (2011),[1] in order to increase clarity of music-based interventions in a more structured way, as requested in the Consolidating Standards for Reporting Clinical Trials (CONSORT), but has been further extended.

A: Intervention Theory Item 2. Theory and/or scientific rationale

Music-based interventions have in general been found beneficial to people with Parkinson's disease (PD).[2,3] Music leads to immediate effects by activating action-related processes, as well as long-term effects such as improved gait in people with PD.[4] The most potent component is *rhythm*, because it acts as an external cue, bypassing the damaged basal ganglia.[4] The Ronnie Gardiner Method (RGM) is a music-based intervention based on auditory rhythm (i.e., beat-based music perceived through hearing) with the goal to improve postural control, enhance gait, and improve cognitive function such as memory. Auditory rhythm given through music influences the kinetic system through synchronisation and adjustments of muscles to the stimuli of music and facilitates movement synchronisation, coordination, and regularisation.[5] The exercises can be performed either sitting down or standing up. While standing, many weight-shifts are involved, challenging anticipatory and reactive postural control. By means of the choice of the symbols and their order, exercises are created to practice starting and stopping, walking forwards and backwards, or stepping aside.

With respect to dual-task performance, RGM is a motor-cognitive intervention that incorporates multiple external cues. Apart from the auditory cues, somatosensory cues through body percussion (e.g., handclaps, stomping with feet, slapping thighs) and visual cues (projected symbols on a screen) are also used.[4] These proposed effect mechanisms are expected to improve motor control and cognitive skills such as concentration, information processing, working memory, and thereby dual-task performance. For information, please visit the official website www.ronniegardinermethod.com.

B: Intervention Content

Each session was initiated with soft stretching movements and breathing exercises, followed by 50 minutes of exercises typical the RGM,[4] and ended with soft classical music. Short breaks were provided between the exercises.

B.1: Person Selecting the Music Item 3a. Music Selection

The music was pre-selected each week by the practitioners based on: a) appropriate tempo (defined by beats per minute, BPM) for the participants; b) different time signatures (mainly 4/4); and c) participants' selections. Efforts were made to choose familiar "feel-good" music, or uplifting music to awaken memories from the participants' adolescence with the potential to influence the atmosphere, mood, and the impact of the exercises in a positive way. Participants were also encouraged to bring favourite music from home or were given opportunities to request any piece of favourite music to optimise involvement and enjoyment.

B.2: Music Item 3b Music

Published music was continuously used. The qualities of the music (e.g., form, melody, harmony, voicing, and tonality) were not considered, as the focus was on using rhythmical music with a steady beat to the exercises. Examples are given below:

**Title:**	**Artist:**	**Tempo:**
Happy together	The Turtles	61 BPM
Say you, say me	Lionel Ritchie	64
Get up, stand up	Peter Tosh	69
Lonely avenue	Ray Charles	72
Layla	Eric Clapton	94
The French march	The Chieftains	108
Wade in the water	Eva Cassidy	112
Billy Jean	Michael Jackson	116

During short breaks and at the end of each session, slow and soft music was used to elicit a relaxed atmosphere, with the addition of a projected picture of nature on the screen to rest the eyes upon.

B.3: Music Delivery Method Item 3c. Music delievery method

Recorded music was used, delivered through speakers placed at one side of the room. This allowed participants to simultaneously attend to the music, the instructions, and the other group members, eliciting attention. The volume was controlled by the interventionists and set based on the participants being able to clearly hear the beat of the music, as well as the instructions. The intention was also to rise physiological arousal levels, adding to the invigorating effect. The volume was immediately lowered upon request if any of the participants expressed discomfort with respect to auditory health and unhealthy sound levels. No decibel level was noted.

B.4: Intervention Materials Item 3d. Materials

No musical instruments or items to create musical sounds were used. Non-musical materials were: audio playback equipment (Onkyo Compact disc player DX-7355), a laptop (HP Elite Book) with output VGA and electrical source, a white cloth, and a connected projector (Logitech). A remote (battery powered) was used to change the slides of the PowerPoint presentation with the note systems.

B.5: Intervention Strategies Item 3e. Intervention Strategy

Certain note systems – referred to as *choreoscores* – with, for RGM unique, red and blue symbols resembling hands and feet were projected on a screen[a]. These symbols work as perceptual cues (colours and shapes). Each symbol is associated with a movement (e.g., stomp with right foot on the floor) and a certain verbal code (e.g., BOOM or CHIC). In this study, 13 out of the existing 19 RGM specific symbols were used. To the rhythm of music, the participants visually read the choreoscores row by row from left to right, while synchronising their motor rhythm with the auditory rhythm and pronouncing the verbal codes loudly beat by beat. Every symbol has its own movement and verbal code to be pronounced while simultaneously performing the movement. When new symbols were introduced, the same pedagogical model was used in order to facilitate learning: first the word was spoken out loud repeatedly, then the motor skills were practiced repeatedly, and last all elements were put together, also repeatedly. Finally, the new symbol was practiced to music at an appropriate tempo. Participants occasionally received handouts with encouragement to repeat the movements at home to facilitate learning.

The exercises were performed either sitting down or standing up. If the movements were experienced as being too challenging, the participants were offered a stationary parallel bar to hold on to for safety. For progression, the note systems were delivered with increasing musical tempo, and/or with more complex symbols and movements.

C: Intervention Delivery Schedule Item 7. Delivery Schedule

Sessions were delivered twice weekly (Mondays and Fridays) for twelve weeks (n= 24). Each session was 60 minutes. Participants were encouraged to practice at home, but without control.

D: Interventionists Item 4. Interventionist

To strengthen arguments for an intervention effect rather than a person effect, two experienced physiotherapists provided the intervention, both with a bachelor's degree and certified RGM practitioners. The certification process typically involves completing three 2-day courses with Mr. Ronnie Gardiner himself or teachers appointed by Mr. Gardiner. Both practitioners had several years of practice from teaching RGM (4 and 10 years, respectively) and of working with people with PD, individually as well as in groups (27 and 19 years). The interventionists both had much experience of

---

[a] Due to publication rights, details of these symbols/movements/codes cannot be described in detail here. However, examples can be found on Google or YouTube when searching for "Ronnie Gardiner Method". Any certified RGM practitioner are welcome to contact the first author (principal investigator) and take part of all note systems used in the intervention on request in order to replicate the intervention.

creating choreoscores suitable for different neurological diseases and age groups. The principal investigator offered meetings regularly to discuss any possible difficulties with the programme.

E: Fidelity Strategies for Treatment Delivery Item 8. Treatment Fidelity

A third qualified RGM practitioner (music therapist) was contracted to check for accuracy, i.e., that the protocol was delivered as intended. This was done at the 11th session.

F: Setting Item 6. Setting

The intervention was delivered in a spacious room at a neurological rehabilitation center in Linköping, Sweden. A stationary parallel bar was placed at one side of the room for those who needed more support during stance.

G: Unit of Delivery

The intervention was delivered in groups of individuals for the potential benefit of social interaction, peer support, and motivation through group accountability. Because of the size of the room, a pre-defined maximum amount of 15 people in the group was decided upon.

References

1. Robb SL, Burns DS, Carpenter JS. Reporting guidelines for music-based interventions. *J Health Psychol.* 2011;16(2):342-352.
2. Zhang S, Liu D, Ye D, Li H, Chen F. Can music-based movement therapy improve motor dysfunction in patients with Parkinson's disease? Systematic review and meta-analysis. *Neurol Sci.* 2017;38(9):1629-1636.
3. de Dreu MJ, van der Wilk AS, Poppe E, Kwakkel G, van Wegen EE. Rehabilitation, exercise therapy and music in patients with Parkinson's disease: a meta-analysis of the effects of music-based movement therapy on walking ability, balance and quality of life. *Parkinsonism Relat Disord.* 2012;18 Suppl 1:S114-119.
4. Pohl P. The Ronnie Gardiner Method: An innovative music-based intervention in neurorehabilitation with focus on Parkinson's disease. *Neurophysio and Rehab.* 2018(1):32-37.
5. Raglio A. Music Therapy Interventions in Parkinson's Disease: The State-of-the-Art. *Front Neurol.* 2015;6:185.
